# Exercise-responsive circTSN as a potential systemic biomarker during COPD rehabilitation

**DOI:** 10.3389/fmed.2025.1735444

**Published:** 2025-12-03

**Authors:** Lei Zhao, Fan Bai, Haizhu Zeng, Rreshma Akter, Huali Zhang, Xiaoxiang Liu

**Affiliations:** 1 Department of Pulmonary and Critical Care Medicine, Shanghai Pudong New Area Gongli Hospital, Shanghai, China; 2 Department of Radiology, First People’s Hospital of Changde, Hunan, China; 3 Program of Bio-Environmental Science, Morgan State University, Baltimore, MD, United States

**Keywords:** chronic obstructive pulmonary disease, circ TSN, pulmonary rehabilitation (PR), exercise training, TSN (Translin), inflammation, cardiopulmonary exercise test (CPET), mean response time (MRT)

## Abstract

**Objective:**

Chronic Obstructive Pulmonary Disease (COPD) features both pulmonary and systemic manifestations. While exercise is a proven therapeutic strategy, the specific non-coding RNAs mediating the systemic adaptive response remain largely unknown. This study aims to identify circTSN as a key circular RNA (circRNA) involved in exercise-induced adaptation in COPD.

**Methods:**

We performed a prospective cohort study using peripheral blood RNA sequencing in COPD patients (*n* = 4) before and after a 12-week exercise intervention, and validated the expression changes with RT-qPCR (*n* = 18). The mechanism of circTSN (hsa-circ_0003789) was investigated using a COPD mouse model, dual-luciferase reporter assays in 293T cells, and functional *in vitro* experiments in BEAS-2B airway epithelial cells.

**Results:**

circTSN expression was significantly upregulated post-exercise in both COPD patients and the mouse model, and this change was decoupled from its host gene, establishing it as an independent molecular marker. Mechanistically, circTSN was found to be cytoplasmically enriched and functioned as a molecular sponge for miR-144-3p. This axis subsequently modulated the expression of key inflammatory genes in BEAS-2B airway epithelial cells, including GATA6, IL-6, IL-1β, and TNF-α, linking the airway epithelium to systemic inflammation. Furthermore, *in vivo*, circTSN and miR-144-3p were inversely correlated, establishing this ceRNA axis as critical for exercise-mediated lung adaptation.

**Conclusion:**

circTSN may serve as a molecular sensor linking changes in the pulmonary environment to the body’s broader adaptive responses during exercise therapy. This discovery offers crucial mechanistic insight into how exercise benefits individuals with COPD, highlighting circTSN as a valuable target for future molecular monitoring and therapeutic strategies.

## 1 Introduction

Chronic obstructive pulmonary disease (COPD) is a leading global health concern, contributing substantially to illness and death worldwide (1). Given the heterogeneity of COPD, despite advances in pharmacological treatments, current options primarily alleviate symptoms without effectively modifying disease progression (2). Previous studies indicate that pulmonary rehabilitation (PR) improves both pulmonary and extrapulmonary function by attenuating airway inflammation, reducing oxidative stress, enhancing skeletal muscle performance (3), and mitigating structural remodeling (4–12). However, the molecular pathways linking exercise interventions with disease modification remain incompletely understood, underscoring the need to identify key biomarkers that can capture these biological responses.

Circular RNAs (circRNAs) are a class of covalently closed, stable, and highly conserved non-coding RNAs that play essential roles in post-transcriptional gene regulation. Owing to their resistance to exonuclease degradation and long half-life (13), circRNAs remain remarkably stable in biological fluids and even during delayed sample processing at 4 °C for up to 48 h. These properties make circRNAs attractive candidates for use as disease biomarkers in human blood and other accessible samples. Dysregulated circRNAs have been linked to lung injury progression (14). Cigarette smoke–induced epithelial injury, and abnormal macrophage activation. For instance, circ_0008833, circ_0025843, and circCANX (15–17) mediate epithelial pyroptosis, ferroptosis, and autophagy, respectively; circSMG6 regulates extracellular matrix turnover (18); m^6^A-circSAV1 (19) modulates oxidative stress through YTHDF1 binding; and circRMRP, circRPL27, circ_0000554, and circADAMTS6, et.al, were related to inflammation via enhancing M2 polarization or glycolytic reprogramming (20–22).

However, most prior studies lacked *in vivo* confirmation. Although several plasma or PBMC-derived circRNAs have been reported to correlate with COPD severity and exacerbation risk (23), these findings were derived from small cohorts and have not been validated in independent, publicly available datasets. Here, we validated an exercise-responsive circTSN (hsa-circ-0003789) using transcriptomic, qPCR, and external bulk RNA-seq analyses, providing clinical evidence of its systemic relevance in COPD rehabilitation. Mechanistically, most reported circRNAs exert their effects through miRNA sponge activity. Furthermore, few studies have investigated whether circRNAs are dynamically regulated during therapeutic interventions such as pulmonary rehabilitation or exercise training, which are known to modulate systemic inflammation and immune function. Identifying exercise-responsive circRNAs could therefore bridge the gap between molecular regulation and clinical rehabilitation outcomes, offering new perspectives for personalized therapy.

Building on this rationale, the present study aimed to explore the role of circTSN, a potential exercise-responsive circRNA, in COPD rehabilitation and to elucidate its potential regulatory mechanism involving the miR-144-3p/GATA6 axis.

## 2 Methods

Eighteen patients diagnosed with COPD were consecutively enrolled from the clinical cohort at Pudong New Area Gongli Hospital (trial registration: ChiCTR2100053232, November 16, 2021). The study was approved by the Ethics Committee of Gongli Hospital, Pudong New Area (Approval No. 2021 [028]), and all participants provided written informed consent.

This was a prospective, two-center study evaluating the effects of a 12-week supervised PR program in patients with COPD. Baseline characteristics were recorded at enrollment. All detailed procedures are described in the Supplementary Materials.

## 3 Results

### 3.1 Pulmonary rehabilitation attenuates body inflammation and reshapes peripheral immune cell composition in COPD patients

**Figure 1:**
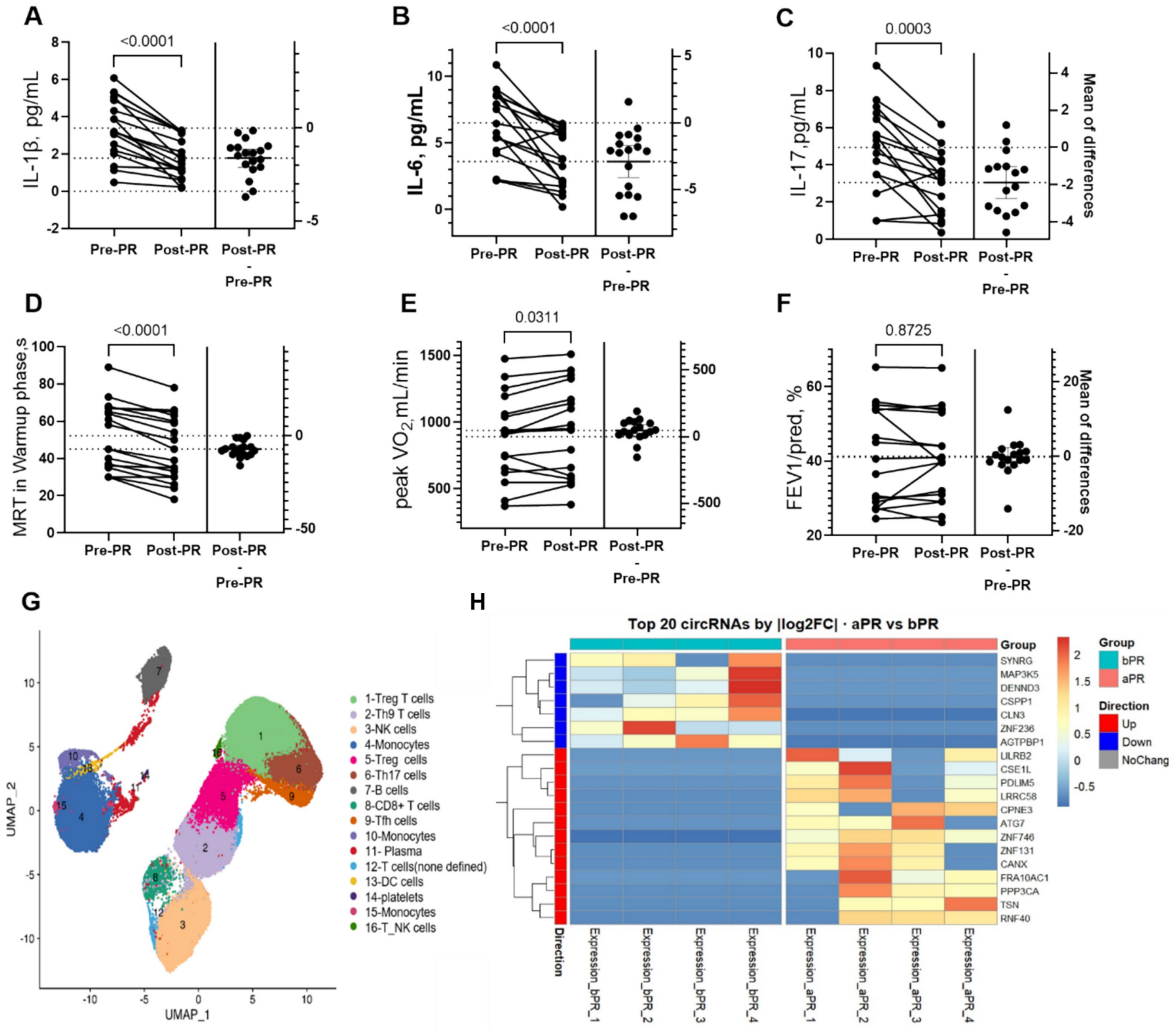
Outcomes following pulmonary rehabilitation in COPD patients. **(A–C)** Serum cytokine levels before (Pre-PR) and after (post-PR) the 12-week pulmonary rehabilitation. Mean differences of Δchanges are shown on the right. **(D–F)** Mean Residence Time (MRT) during warmup phase, Peak VO_2_, and FEV1%predicted before and after PR. *p*-values are from paired tests. **(G)** scRNA-seq showing cell subset redistribution. **(H)** Heatmap showing the top 20 differentially expressed circRNAs (log_2_FC > 1, adjusted *p* < 0.05) between patients who benefited from PR (aPR) and their baseline (bPR).

Baseline characteristics of the 18 recruited COPD patients are summarized in Supplementary Table S1. Following the intervention, plasma IL-6, IL-1β, and IL-17 levels significantly decreased (Figures 1A–C), accompanied by increased ventilatory efficiency (Figures 1D,E) (ΔMRT, ΔVO₂ peak) (*p* < 0.05). In contrast, forced expiratory volume in 1 second (FEV₁ % predicted) showed no significant change after training (Figure 1F). These results indicate that exercise rehabilitation improved systemic inflammation and cardiopulmonary efficiency (24), even though static lung capacity remained largely unchanged.

Using single-cell RNA sequencing (scRNA-seq) of whole-blood cells from a subset of patients (*n* = 3) and age-matched healthy controls (*n* = 3), we identified 11 transcriptionally distinct cell clusters spanning major immune lineages (Figure 1G). Notably, T-cell clusters (Clusters 2 and 5) were reduced in COPD patients compared with healthy controls, with proportions decreasing by approximately 10%–15%. In contrast, pulmonary rehabilitation partially reversed this trend by about 5%, indicating a moderate recovery of peripheral immune homeostasis. (Supplementary Figure S1). Flow-cytometric analysis yielded consistent findings (data not shown). Further comparison from bulk RNA-seq from peripheral blood leukocytes between post-rehabilitation (aPR) and pre-rehabilitation (bPR) samples revealed 2,064 differentially expressed circular RNAs (circRNAs), including 676 upregulated and 1,388 downregulated transcripts (Figure 1H, right panel). These results indicate that specific transcriptional and post-transcriptional reprogramming events in circulating immune cells may contribute to the observed clinical improvements following pulmonary rehabilitation.

### 3.2 circTSN is upregulated following exercise and conserved across human and mouse COPD models, correlating with systemic inflammation

**Figure 2:**
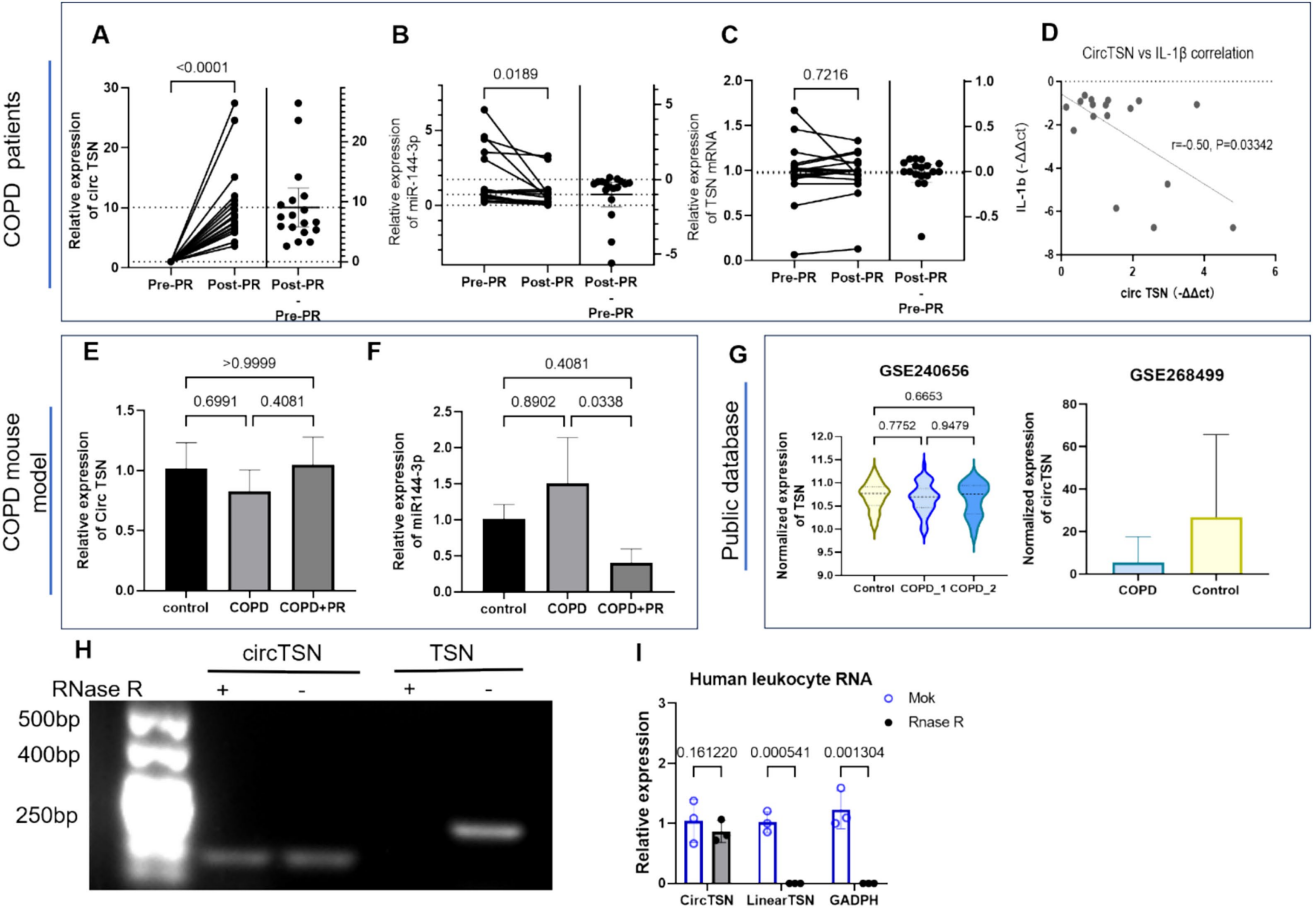
Relative expression of circ TSN and miR-144-3p in peripheral leukocytes from COPD patients and lung tissues of COPD mice. **(A–C)** circTSN, miR-144-3p, and linear TSN relative expression in peripheral leukocytes from COPD patients before (Pre-PR) and after pulmonary rehabilitation (Post-PR). Data were analyzed using ΔCt values. For clarity, fold change (2^−ΔΔCt^) is shown in the figures. **(D)** The scatter plot shows post–pre changes in circTSN versus cytokine IL-1β (*r* = −0.50, *p* = 0.033). **(E,F)** circTSN and miR-144-3p expression in lung tissue from the elastase-induced COPD mouse model, including control, COPD, and COPD + PR (COPD mice with pulmonary rehabilitation) groups. Data are presented as mean ± SD (*n* = 3 per group). **(H,I)** RNase R digestion confirming circularity of circTSN in BEAS-2B cells. **(G)** External validation (GSE240656, GSE268499) reveals a reduced trend of circTSN in COPD, with no significant change in TSN mRNA.

circTSN expression levels in COPD patients before and after PR were validated by RT-qPCR, confirming our initial sequencing findings (25): CircTSN was significantly upregulated after the 12-week PR program (Figure 2A). At the same time, its predicted target, miR-144-3p, showed a significant reduction (*p* = 0.018) (Figure 2B). Notably, the host linear TSN transcript remained entirely unchanged (Figure 2C), indicating that circTSN modulation is decoupled from linear transcription and driven by independent regulatory mechanisms. Furthermore, correlation analysis showed that the exercise-induced increase in circTSN was accompanied by a decrease in the systemic inflammatory marker IL-1β (*r* = −0.50, *p* = 0.033, Figure 2D) and an increase in body oxygen consumption (Table 1), suggesting a direct link between circTSN upregulation and relief of inflammation after rehabilitation. The stability of circTSN as a circulating biomarker was assessed via RNase R digestion in human leukocyte RNA, confirming that circTSN exhibits robust resistance to enzymatic degradation, unlike its linear host gene and GAPDH (Figures 2H,I). To confirm this molecular response *in vivo*, we utilized an elastase-induced COPD mouse model comprising control, COPD, and exercise groups (*n* = 3 mice per group). In line with the human findings, circTSN expression in lung tissues showed a decreasing trend in COPD mice and a partial restoration following treadmill exercise, whereas miR-144-3p exhibited the opposite pattern (Figures 2E,F). Although these changes did not reach statistical significance, the directionality of expression was consistent with that observed in patient samples, suggesting a similar molecular response to pulmonary rehabilitation in mice. This finding was further supported by external validation using independent, publicly available datasets (GSE240656 and GSE268499), which consistently showed reduced circTSN expression in COPD patients and no difference between the two groups (Figure 2G).

**Table 1:** Correlation between changes in circTSN expression and inflammatory or cardiopulmonary parameters in COPD patients.

Outcome	Method	*r*	*p*	q(FDR)
ΔIL-1β	Spearman	−0.5028	0.0334	0.0835
ΔIL-6	Pearson	0.2689	0.2806	0.35075
ΔVE/VCO₂ slope		−0.05503	0.8338	0.8855
ΔVO_₂_peak		0.5213	0.0265	0.0835
MRT in rest		−0.3944	0.1053	0.1755

Given the small sample size, the statistical power to detect significant correlations is limited. False discovery rate (FDR) adjustments were applied for exploratory purposes only, and unadjusted *p*-values should be interpreted with caution.

These data consistently identify circTSN as an exercise-responsive circulating RNA whose expression is independently regulated. Its robust stability and inverse correlation with systemic inflammation strongly support its potential as a systemic biomarker of physiological adaptation to pulmonary rehabilitation. Based on this established molecular association, we next explored whether circTSN exerts its anti-inflammatory effect through a specific miRNA-sponging mechanism.

### 3.3 circTSN directly interacts with miR-144-3p and regulates the downstream target GATA6 to modulate inflammatory signaling

**Figure 3:**
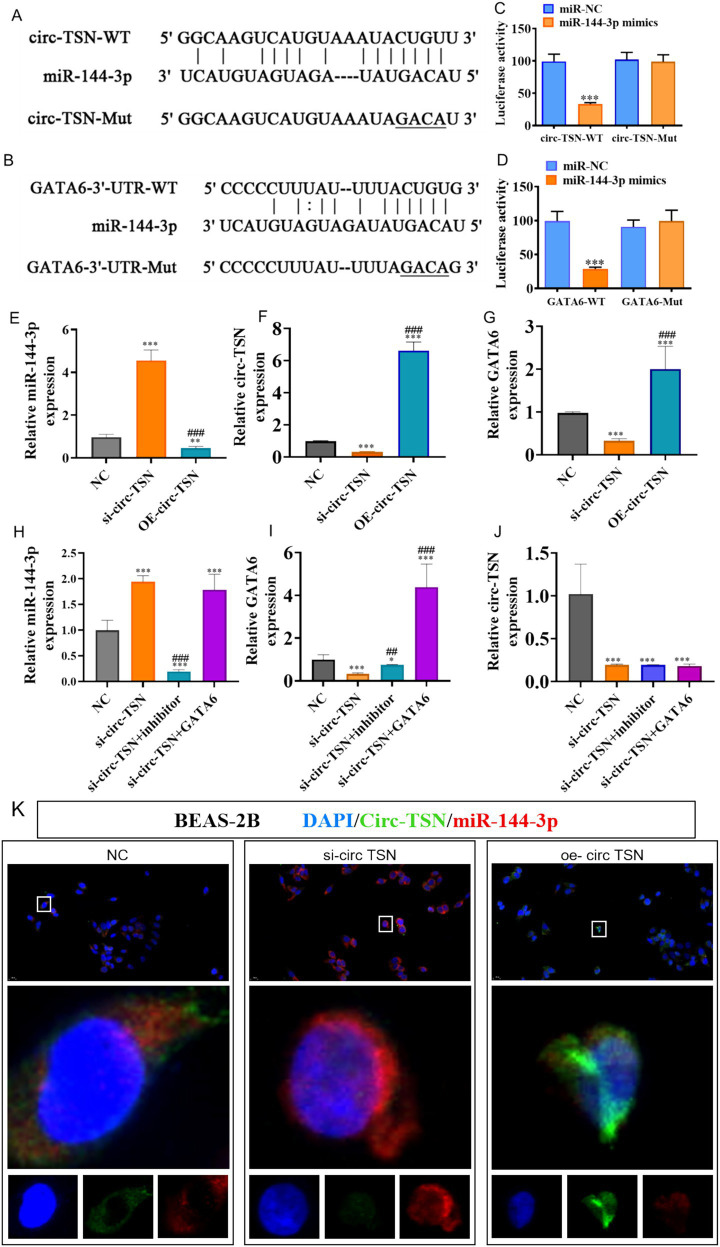
circTSN functions as a molecular sponge for miR-144-3p to regulate GATA6 expression in BEAS-2B cells. **(A,B)** Dual-luciferase reporter assays confirming direct binding of miR-144-3p to circTSN **(A)** and the GATA6 3′-UTR **(B)**. Mutation of the predicted binding sites abolished the inhibitory effect of miR-144-3p on luciferase activity. **(C–J)** Expression analysis of circTSN, miR-144-3p, and GATA6 in BEAS-2B cells following circTSN overexpression (OE), knockdown (si-circTSN), or miR-144-3p inhibitor. **(K)** Fluorescence *in situ* hybridization (FISH) images showing subcellular co-localization of circTSN (green) and miR-144-3p (red) in BEAS-2B cells under NC, OE, and si-circTSN conditions. Nuclei are stained with DAPI (blue). Co-localization in the cytoplasm supports their interaction.

Given that circTSN expression was closely associated with inflammatory responses in COPD, we explored its potential molecular mechanism. Bioinformatic prediction indicated that circTSN harbors a conserved binding site for miR-144-3p (Figure 3A), a miRNA previously implicated in epithelial injury and inflammation. Dual-fluorescence *in situ* hybridization (FISH) revealed that both circTSN and miR-144-3p were predominantly localized in the cytoplasm of BEAS-2B cells (Figure 3K), with clear co-localization signals, suggesting a potential direct interaction.

To verify this interaction, dual-luciferase reporter assays were performed. Co-transfection of miR-144-3p mimic significantly decreased luciferase activity of the circTSN-WT reporter but not the Mut construct, confirming that circTSN directly binds to miR-144-3p. Similarly, miR-144-3p was shown to bind to the 3′-UTR of GATA6, and mutation of the predicted site abolished this inhibitory effect, indicating that GATA6 is a downstream target of miR-144-3p (Figures 3C,D). To further confirm the functional relationship within the circTSN/miR-144-3p/GATA6 axis, we examined their expression changes under different modulation conditions in BEAS-2B cells (Figures 3E–G). circTSN knockdown (si-circTSN) led to a significant increase in miR-144-3p expression and a marked reduction in both circTSN and GATA6 levels. In contrast, circTSN overexpression (OE-circTSN) produced the opposite effects, indicating that circTSN negatively regulates miR-144-3p and positively regulates GATA6.

To explore whether this effect is mediated through miR-144-3p, we introduced a miR-144-3p inhibitor and a GATA6 overexpression plasmid in circTSN-silenced cells. As shown in Figures 3H–J, inhibition of miR-144-3p or forced expression of GATA6 partially rescued the reduction of GATA6 expression induced by circTSN knockdown, while circTSN levels remained unchanged.

## 4 Discussion

In this study, we observed that circTSN expression in peripheral blood was closely associated with inflammatory status in patients with COPD. Exercise training significantly reduced systemic levels of IL-6, IL-1β, and IL-17, while circTSN expression showed a negative correlation with them. These findings suggest that circTSN may be involved in inflammation-related regulation rather than being a mere bystander molecule. These pilot *in vivo* data support the feasibility of the COPD mouse model for exploring exercise-responsive regulatory mechanisms, but larger sample sizes will be required to confirm the observed trends.

Previous studies have shown that multiple circRNAs, such as circSMG6, circCANX, and circADAMTS6, participate in airway inflammation, epithelial injury, and macrophage polarization. Consistent with these reports, our results indicate that circTSN is correlated with systemic inflammation in COPD. To further explore how circTSN were involved in COPD, we examined its downstream interactions in BEAS-2B epithelial cells. Dual-luciferase and FISH assays confirmed that circTSN directly binds to miR-144-3p, while miR-144-3p targets the 3′-UTR of GATA6. Experiments involving overexpression and silencing of circTSN demonstrated reciprocal regulation among these molecules, suggesting that circTSN modulates the miR-144-3p–GATA6 signaling axis *in vitro*. The anti-inflammatory trend observed in epithelial cells was consistent with the clinical data, reinforcing the link between circTSN expression and inflammatory regulation.

Accumulating evidence identifies miR-144-3p as a key post-transcriptional regulator involved in the inflammatory pathogenesis of chronic obstructive pulmonary disease (COPD). It is frequently elevated in both the airway epithelium and circulating leukocytes of COPD patients, where it intensifies inflammation by repressing multiple mRNAs associated with epithelial integrity, antioxidant defense, and tissue repair (26). Although miR-144-3p has been reported to interact with GATA3 and GATA4 (27) in non-pulmonary systems, GATA6 is the principal GATA family member expressed in airway epithelial cells, orchestrating differentiation, barrier renewal, and immune homeostasis. Prior studies show that GATA6 modulates epithelial inflammation and contributes to alveolar regeneration after injury (28). Our results support a circTSN/miR-144-3p/GATA6 regulatory model, in which circTSN sponges miR-144-3p, thereby alleviating the repression of GATA6 and promoting epithelial repair while limiting inflammatory signaling. The anti-inflammatory effects observed after exercise training may therefore reflect enhanced epithelial resilience mediated through this axis. These findings highlight circTSN as a potential inflammation-associated circular RNA contributing to the molecular benefits of pulmonary rehabilitation in COPD and represent a promising target linking epithelial repair with systemic immune modulation.

Beyond molecular observations, the physiological findings further illustrate the systemic benefits of pulmonary rehabilitation. Some patients with COPD were unable to complete maximal cardiopulmonary exercise testing because of ventilatory limitation or fatigue. Nevertheless, parameters recorded during the warm-up phase, particularly MRT, provided an objective reflection of improved oxygen delivery and utilization after training. A shorter MRT indicates enhanced cardiopulmonary coordination and reduced oxygen deficiency. The improvement in MRT may also reflect attenuation of systemic inflammation, as improved oxygen dynamics could weaken the reciprocal loop between hypoxia and inflammation that is typical of COPD.

This study has several limitations. Although both bulk and single-cell transcriptomic data were analyzed to provide molecular context, functional validation was primarily performed in BEAS-2B epithelial cells, which may not fully reflect *in vivo* airway conditions. The clinical findings were based on peripheral blood rather than airway tissue, and therefore, the direct relationship between circTSN and local airway inflammation remains to be clarified. Moreover, although the circTSN–miR-144-3p–GATA6 interaction was verified *in vitro*, its causal role in the anti-inflammatory response induced by exercise training has yet to be confirmed.

Future studies should extend these findings by validating circTSN expression in airway samples and animal models of COPD. Integrating multi-omics approaches and functional assays will be crucial in determining whether circTSN acts as a key regulator or as a circulating biomarker reflecting the inflammatory state during rehabilitation.

## 5 Conclusion

In summary, circTSN expression was negatively associated with systemic inflammation in patients with COPD. Cellular experiments demonstrated that circTSN interacts with miR-144-3p and regulates GATA6 expression, forming a regulatory pathway that may modulate epithelial inflammatory activity. These results provide a new mechanistic link between molecular regulation and the clinical benefits of exercise rehabilitation. circTSN may serve as a potential biomarker and therapeutic target for improving inflammation control and recovery outcomes in COPD.

## Data Availability

Raw and processed RNA sequencing data generated in this study have been deposited in the NCBI Gene Expression Omnibus GSE235174. De-identified CT and ultrasound imaging data supporting this work have been deposited in Zenodo (https://zenodo.org/records/17427212).
