# Supplementary material for: Exercise-responsive circTSN as a potential systemic biomarker during COPD rehabilitation
Source: Front Med (Lausanne). 2025 Dec 3;12:1735444. doi: 10.3389/fmed.2025.1735444 (PMC12708264; doi:10.3389/fmed.2025.1735444)
Supplement: Supplementary file 1 [file Supplementary_file_1.docx]

**Supplementary Material**

**Exercise-Responsive circTSN as a Potential Systemic Biomarker During COPD Rehabilitation**

Lei Zhao^1‡^ , Bai Fan^2‡^, Rreshma akter^3^, Huali Zhang^1^, Haizhu Zeng^1^, Xiaoxiang Liu^2*^

1. Department of Pulmonary and Critical Care Medicine, Shanghai Pudong New Area Gongli Hospital, 219 MiaoPu Road, Shanghai 200315, P.R.China.
2. Department of Radiology, First People's Hospital of Changde,818 Renming Road, Hunan 415000, P.R.China.
3. Program of Bio-Environmental Science, Morgan State University, MD.21251, USA

^‡^ These authors contributed equally to this work.

^*^Corresponding authors

Xiaoxiang Liu, Department of Radiology, First People's Hospital of Changde,818 Renming Road, Hunan 415000, P.R.China.

Tel: 86+15080656829

E-mail: 791392527@qq.com

1. **Inclusion criteria**

Diagnosis was established according to the Global Initiative for Chronic Obstructive Lung Disease (GOLD) criteria. Patients with clinically significant cardiac impairment, active infectious diseases (such as hepatitis or tuberculosis), or exercise contraindications, including neurological or psychiatric illness, were excluded.

Pulmonary rehabilitation protocol

1. **Exercise intervention**

Exercise training was performed using a cycle ergometer (Qianjing 20003, China). The first three days were dedicated to adaptation, during which workloads were determined individually based on cardiopulmonary exercise testing (CPET) results and continuous functional capacity assessment. From the fourth day onward, participants entered a 12-week supervised training program. Each training day consisted of approximately one hour of cycling, divided into three to four shorter sessions, depending on individual tolerance, and was scheduled five days a week. Exercise intensity was tailored to a moderate level based on CPET-derived parameters. The Δ50% workload (±10 W) was calculated using a formula initially proposed by Professor Xingguo Sun and described in our previous publication.

1. **Clinical outcomes**

Clinical assessments were performed at baseline and after 12 weeks of training. Exercise capacity was measured by the 6-minute walk distance (6MWD) and cardiopulmonary exercise testing (CPET), with peak VO₂, anaerobic threshold, and maximal workload recorded. Symptoms and health status were evaluated using the Modified Medical Research Council (mMRC) scale for dyspnea and the COPD Assessment Test (CAT) for overall symptom burden.

1. **RNA extraction and scRNA-seq, Bulk RNA-seq**

At baseline and after rehabilitation, 3 mL of peripheral blood was collected from participants with COPD and matched controls into EDTA tubes. Leukocytes were isolated within 2h by density gradient centrifugation, and total RNA was extracted using TRIzol reagent (Invitrogen, USA). RNA integrity and purity were assessed with a NanoDrop spectrophotometer (Thermo Fisher, USA). For transcriptome profiling, 3 μg of total RNA per sample was used for library construction with the KAPA Stranded RNA-Seq Kit (Roche) and sequenced on the Illumina HiSeq 4000 platform (Aksomics, Shanghai). RNA sequencing and bioinformatics analyses were performed by OE Biotech (Shanghai).

1. **RT-qPCR**

For validation assays, total RNA was isolated from peripheral blood leukocytes, cultured cells, and murine lung tissues using TRIzol reagent or the MolPure® Blood RNA Kit (Yeasen Biotechnology (Shanghai) Co., Ltd.19241ES50) according to the manufacturer’s instructions. 1 μg of total RNA was reverse-transcribed using the Evo M-MLV Reverse Transcription System (Accurate Biotechnology, Hunan, China; Cat. AG11706) with random hexamers. circTSN was quantified by qPCR using divergent primers spanning the back-splice junction. GAPDH was used as an internal control for mRNA and circRNA. Mature miRNAs were reverse-transcribed using the Mir-X™ miRNA First-Strand Synthesis Kit (Takara, Japan,638313) with miRNA-specific primers; U6 served as the endogenous control. Quantitative PCR was performed on a SLAN-96S real-time PCR platform (Shanghai Hongshi, China) using SYBR Green Pro Taq HS Premixed qPCR Kit (with ROX) (Accurate Biotechnology, Hunan, China; Cat. No. AG11718). Relative transcript abundance was determined by the 2^−ΔΔCt method in accordance with MIQE guidelines. All experiments were performed in quadruplicate to ensure reproducibility. Primer sequences are listed in Supplementary Table S2.

1. **RNase R treatment**

Total RNA (2 µg) from peripheral blood leukocytes was incubated at 37 °C for 20 min with or without RNase R (5 U/µg RNA; Yeasen Biotechnology (Shanghai) Co., Ltd. 14606ES72) in the supplied buffer. RNA was purified using the RNeasy MinElute Cleanup Kit (Qiagen) and eluted in nuclease-free water. Equal RNA inputs from treated and untreated aliquots were reverse-transcribed and quantified by qRT-PCR (see RT-qPCR). Divergent primers spanning the back-splice junction were used for circTSN, and convergent primers for linear TSN.

1. **Fluorescence In Situ Hybridization (FISH) and Co-localization Assay**

BEAS-2B cells were seeded onto sterile glass coverslips in 24-well plates at a density of 3 × 10⁴ cells/well and allowed to adhere overnight. FISH was performed using FAM-labeled circTSN probes (green, targeting the back-splice junction; RiboBio, Guangzhou, China; custom-synthesized) and Cy3-labeled miR-144-3p LNA probes (red; RiboBio, Guangzhou, China; Cat. No. miR20006397-1-1) according to the manufacturer’s protocol. Cells were fixed with 4% paraformaldehyde in PBS for 15 min at room temperature, followed by permeabilization with 0.1% Triton X-100 (Sigma-Aldrich, St. Louis, MO, USA; Cat. No. T8787) for 10 min. Probes were diluted to a working concentration of 100 nM in hybridization buffer (50% formamide, 2× SSC; supplied with the RiboBio kit) and hybridized overnight at 37 °C in a humidified chamber. Following stringent washing, nuclei were counterstained with DAPI (Beyotime Biotechnology, Shanghai, China; Cat. No. C1002, 1 µg/mL, 5 min) and mounted with anti-fade mounting medium (Thermo Fisher Scientific, Waltham, MA, USA; Cat. No. P36930). Fluorescence signals were observed and captured using a Leica TCS SP8 confocal fluorescence microscope (Wetzlar, Germany) equipped with a 63× oil-immersion objective. Images were acquired under identical exposure settings, processed with LAS X software, and presented with a 20 µm scale bar.

1. **Animal model and experimental procedures**

Male BALB/c mice (8 weeks old, 24–25 g) were purchased from Shanghai Jihui Laboratory Animal Care Co., Ltd. (Shanghai, China) and housed under specific pathogen-free (SPF) conditions (23 ± 2 °C; 12-h light/dark cycle) with free access to standard chow and water. Mice were acclimated for one week before experimentation. A total of 30 mice were randomly divided into three groups: control, COPD, and COPD + exercise (n = 10 per group).To establish the COPD model, mice received a single intranasal instillation of 3 U porcine pancreatic elastase (Sigma-Aldrich, USA; 0.3 mg/mL in sterile saline) under light isoflurane anesthesia on Day 0. D14Evaluations were performed on Day 21 after elastase administration, corresponding to the stage when emphysematous changes become evident. After confirmation of successful modeling, mice in the exercise group underwent a treadmill training program consisting of a 5-minute warm-up followed by 30 minutes at 18 m/min (0° incline), 5 days per week for 4 weeks ^1^. At the end of the experiment, mice were anesthetized with 2.0% isoflurane and euthanized by cervical dislocation; the absence of respiration and reflexes confirmed death.

1. **Statistical Analysis**

Quantitative data are expressed as mean ± SD or median [IQR], and categorical variables as counts. Data normality was assessed using the Shapiro–Wilk test. Depending on distribution, group comparisons were performed with paired two-tailed t-tests or Mann–Whitney/Wilcoxon tests; for three or more groups, one-way ANOVA with Bonferroni correction or Kruskal–Wallis test was applied. Correlations were analyzed using Spearman’s coefficient. A p < 0.05 was considered statistically significant. Bioinformatic analyses were performed in R (v4.3.3) using DESeq2 for differential expression. Genes with |log₂ fold change| ≥ 1 and adjusted p < 0.05 were regarded as significant.

|  | **Present COPD cohort**  **(n=18)** | | **GSE 240656 selected**  **(COPD n=77)** | | | **GSE 240656 selected**  **（Control n=169）** |
| --- | --- | --- | --- | --- | --- | --- |
| ***Demographic data*** | | | | | | |
| Sex （male/female） | 16/2 | | 65/12 |  | 142/27 | |
| Age, years | 65.28士5.22 | | 76.00士10.9 | | 64.77士13.44 | |
| BMI, Kg·m2 | 22.23士4.89 | | 23.47士3.51 | | 24.57士3.28 | |
| ***Smoking History*** | | | | | | |
| Current smoker(n) | 2 | | 18 | | 74 | |
| Ex-smoker(n) | 16 | | 48 | | 71 | |
| Smoking, pack-years | 19.82士14.84 | | 23.09士19.76 | | 31.43士22.32 | |
| ***Lung Function*** | | | | | | |
| FEV1(%predicted) | 39.18士16.79 | | / | | / | |
| FEV1 | 48.00士14.10 | | 1.53士0.44 | | 2.24士0.85 | |
| Dlco,/pred,% | 50.10士21.09 | | NA | | NA | |
| ***Complication*** | | | | | | |
| Hypertension/coronary artery disease | | 10 | 0 | | 15 | |
| Osteoporosis | | 6 | NA | | NA | |
| DM | | 0 | 13 | | 46 | |
| Lower limb vascular plaque | | 10 | NA | | NA | |
| ***Exercise performance*** | | | | | | |
| 6-min walk distance (m) | 285.6士79.58 | | NA | | NA | |
| ***Blood cell count(×10⁹/L)*** | | | | | | |
| WBC count | 6.78士1.54 | | 6.81士2.08 | | 10.26士9.363 | |
| Neutrophil percent | 62.48士9.93 | | 58.90士10.17 | | 50.10士21.09 | |

**Results:**

**Table S1: Baseline Characteristics of enrolled patients.**

**Table S2: RT-qPCR Primers used in this study**

| **Primer** | **Sequence** |
| --- | --- |
| Hsa-circ_0003789 forward primer | ACCATGGAGCTTCTTGCCAG |
| Hsa-circ_0003789 reverse primer | CTGCCGAATTCTTGCCAACT |
| hsa-mir-144-3p-RT | GTCGTATCCAGTGCAGGGTCCGAGGTATTCGCACTGGATACGACAGTACA |
| hsa-mir-144-3p  forward primer | GCGCGCGTACAGTATAGATGA |
| hsa-mir-144-3p  reverse primer | AGTGCAGGGTCCGAGGTATT |
| mmu_circ_0008085(TSN)  forward primer | TGGTCTTCCTGGCAGCATTT |
| mmu_circ_0008085(TSN)  forward primer | ACCCTTTTTCCCGATCTGGT |
| mmu-mir-144-3p-RT | GTCGTATCCAGTGCAGGGTCCGAGGTATTCGCACTGGATACGACACTTAC |
| mmu-mir-144-3p  forward primer | GCGCGGGATATCATCATATACT |
| mmu-mir-144-3p  reverse primer | AGTGCAGGGTCCGAGGTATT |


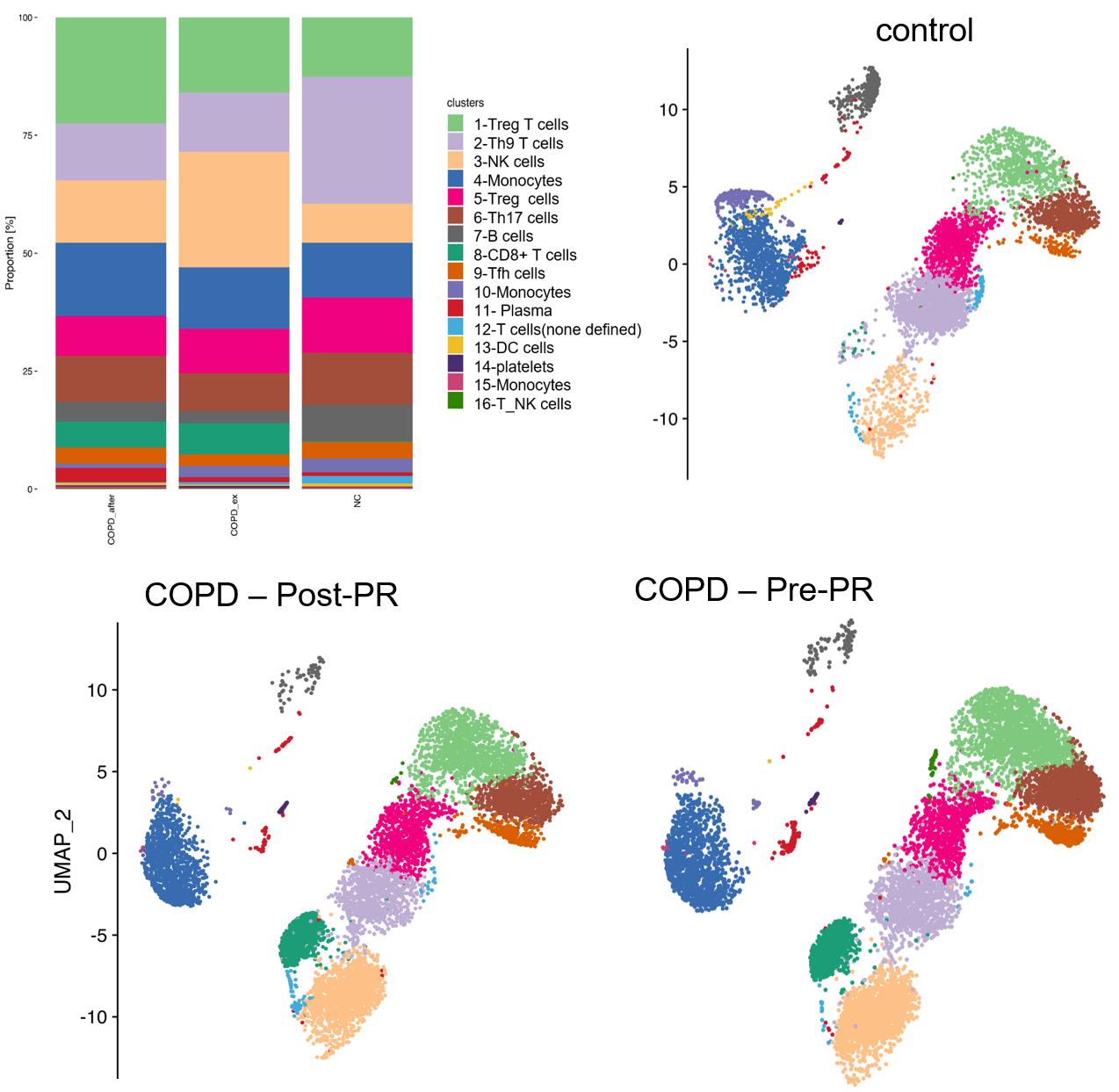
Figure S1 **Single-cell transcriptomic landscape of peripheral blood from healthy individuals, and COPD patients before and after exercise training**

Uniform manifold approximation and projection (UMAP) plots show single-cell RNA sequencing data from the whole blood of healthy individuals, and CODP patients before and after exercise. Eleven major cell clusters were identified, including T cells, B cells, NK cells, monocytes, dendritic cells, and platelets. The overall cell composition remained stable, with mild transcriptional changes related to immune and metabolic activity after exercise.

References:

1. Zeng H, Liu X, Liu P, et al. Exercise’s protective role in chronic obstructive pulmonary disease via modulation of M1 macrophage phenotype through the miR-124-3p/ERN1 axis. *Science Progress* 2025; 108: 00368504251360892.
